# Supplementary material for: Association of Hepatobiliary Phase of Gadoxetic-Acid-Enhanced MRI Imaging with Immune Microenvironment and Response to Atezolizumab Plus Bevacizumab Treatment
Source: Cancers (Basel). 2023 Aug 24;15(17):4234. doi: 10.3390/cancers15174234 (PMC10486496; doi:10.3390/cancers15174234)
Supplement: Supplementary file 1 [file cancers-15-04234-s001.zip › Supplementary Figures_PDF.pdf]

Supplementary Figure S1

a

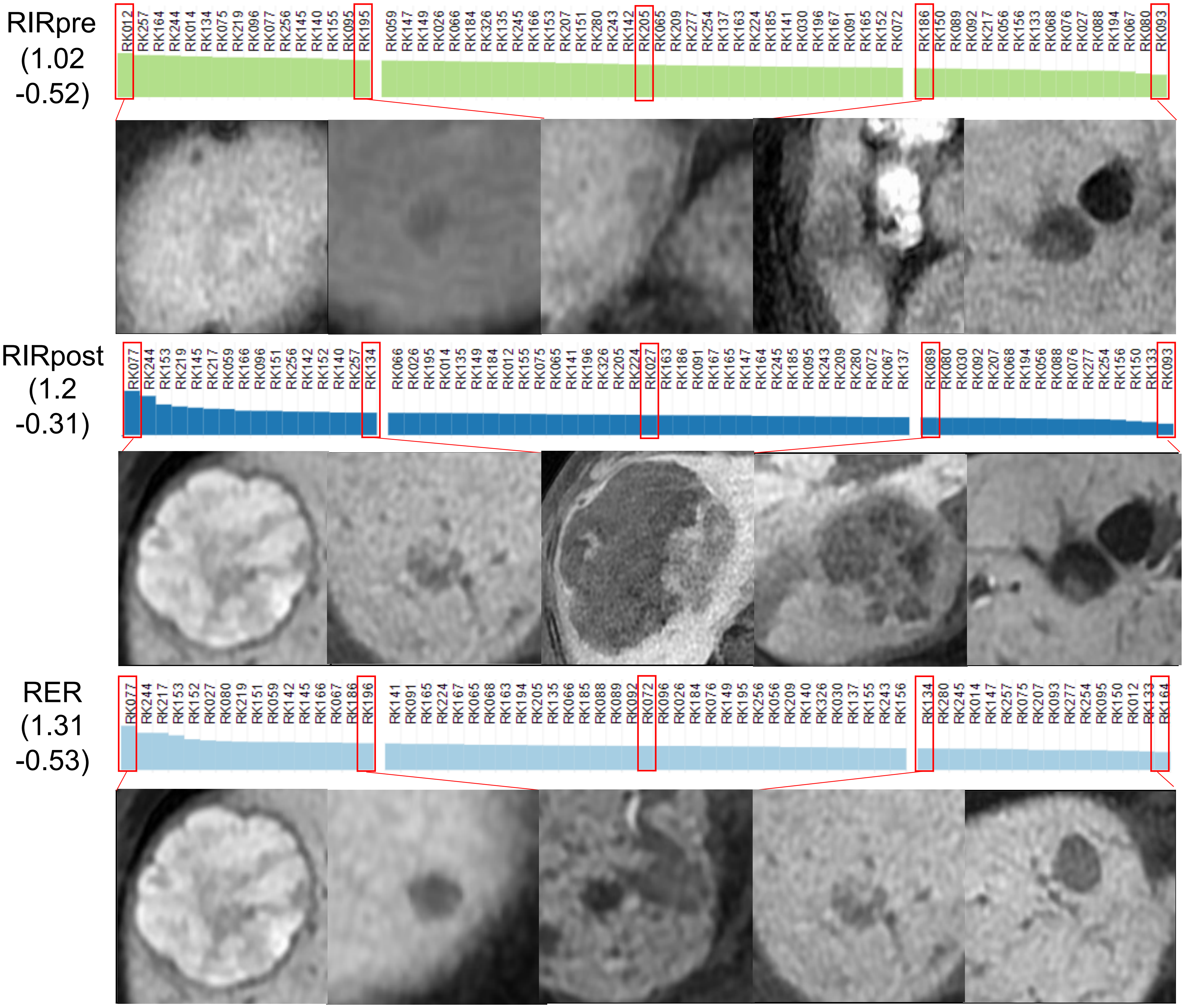

b

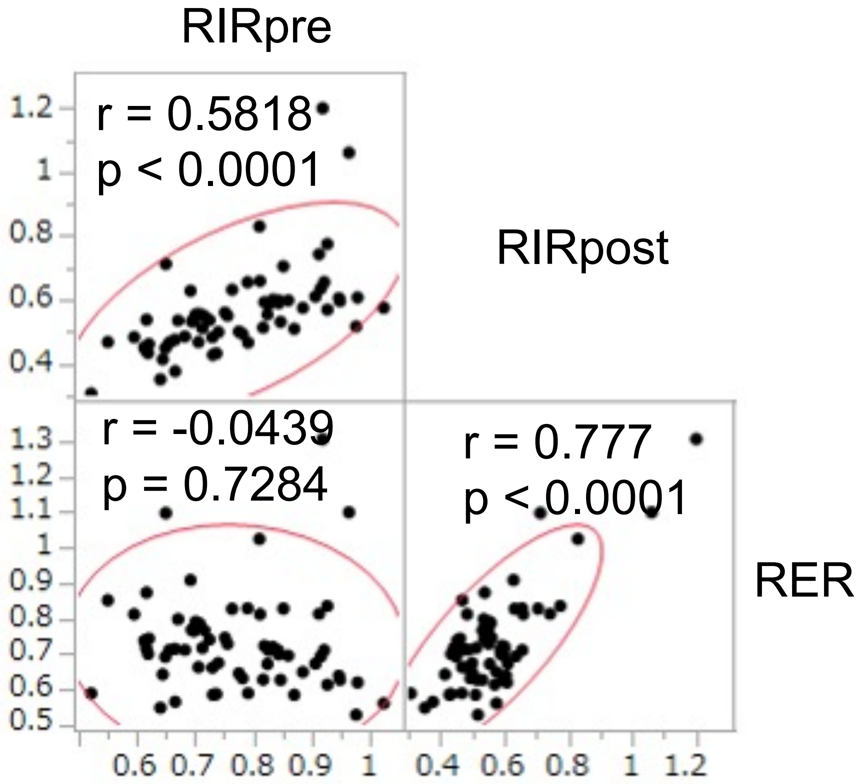

c

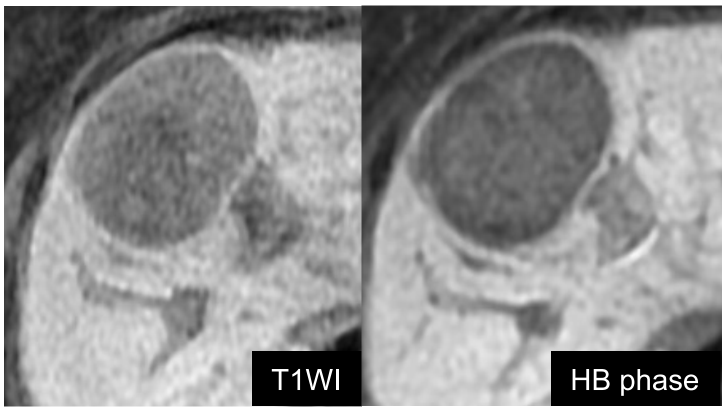

Supplementary Figure S2

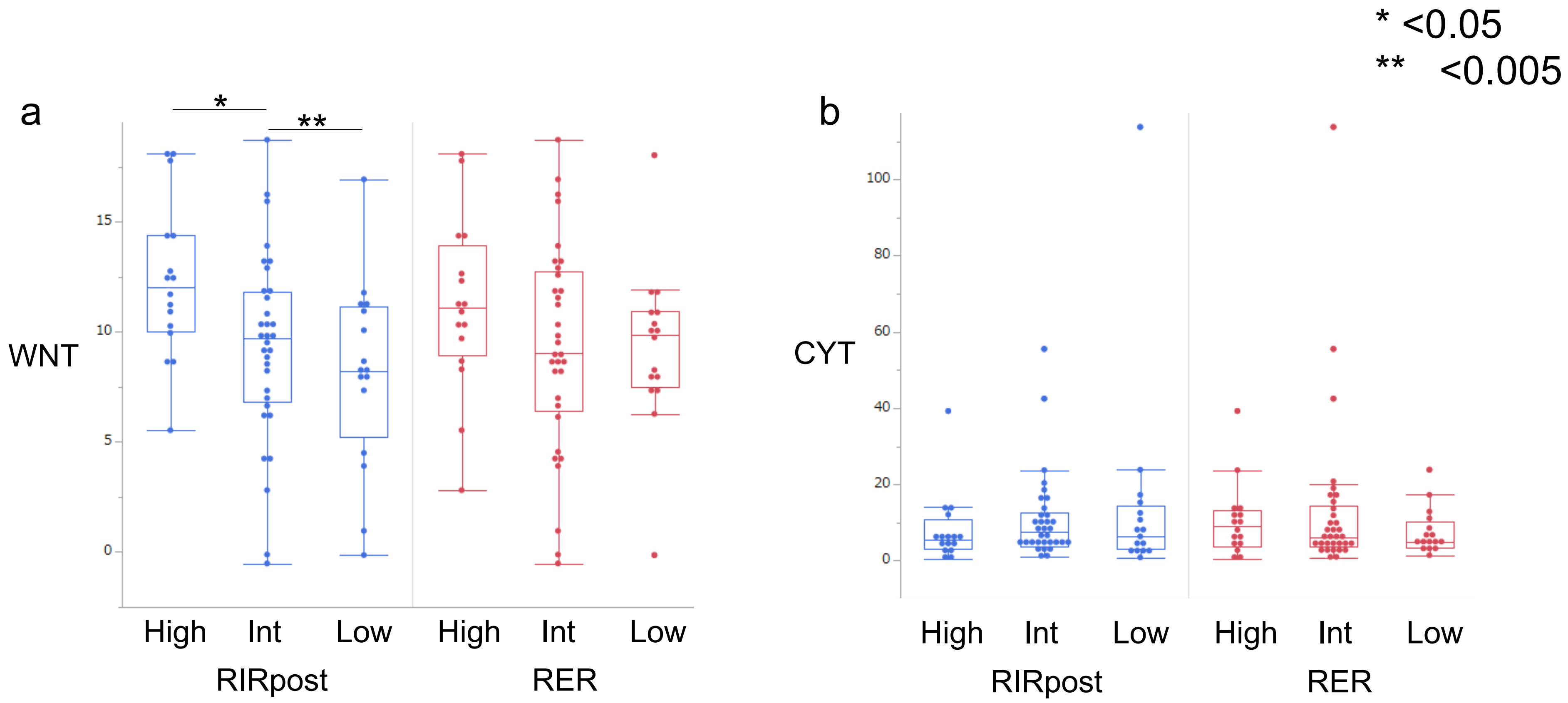

Supplementary Figure S3

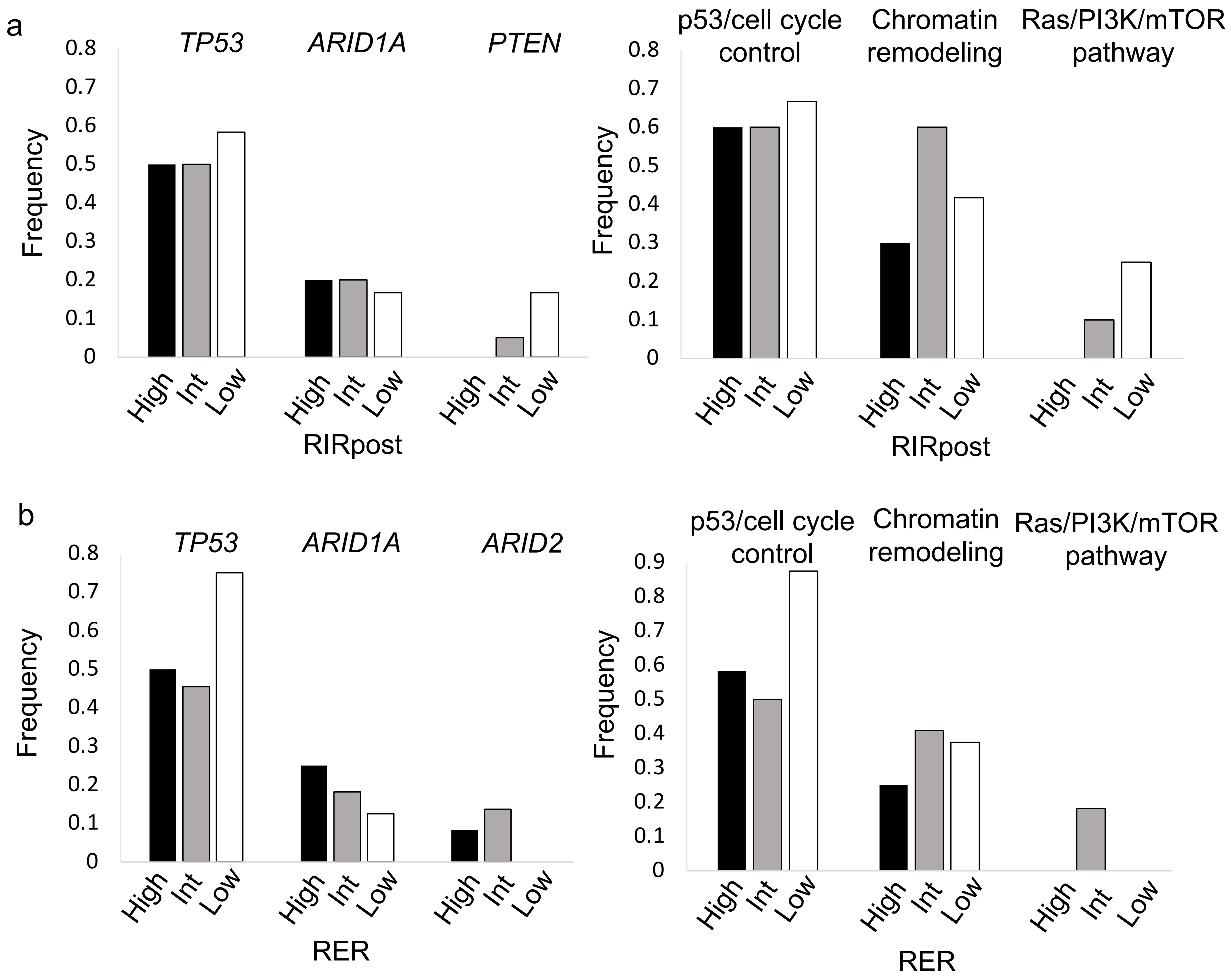

Supplementary Figure S4

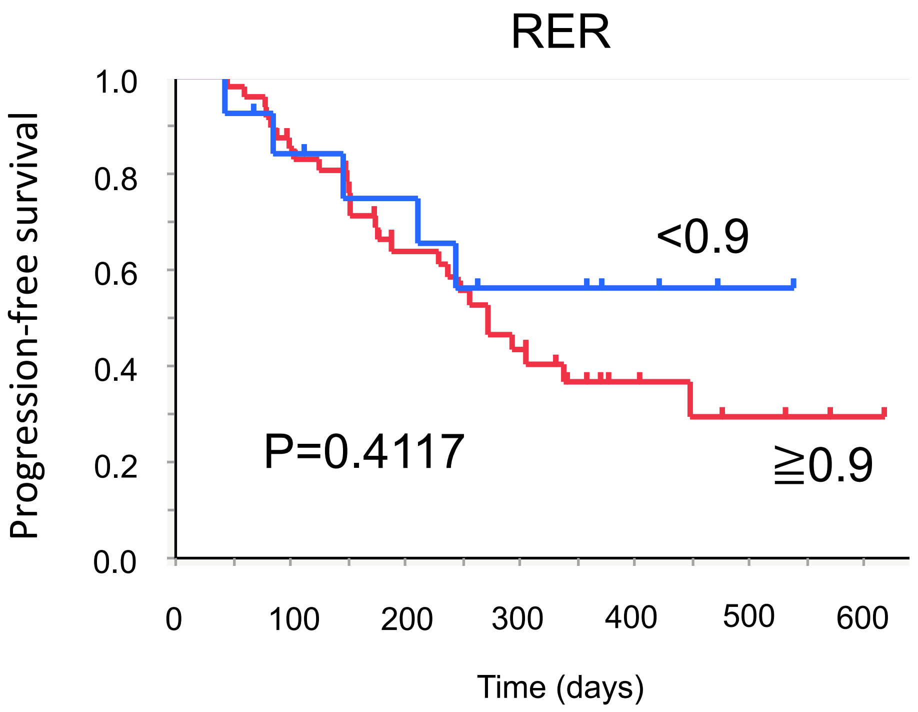

Number at risk							
≧0.9	13	11	9	6	4	2	1
<0.9	47	39	26	15	7	4	2
